# Impact of Methylmercury and Other Heavy Metals Exposure on Neurocognitive Function in Children Aged 7 Years: Study Protocol of the Follow-up

**DOI:** 10.2188/jea.JE20190284

**Published:** 2021-02-05

**Authors:** Liza Vecchi Brumatti, Valentina Rosolen, Marika Mariuz, Elisa Piscianz, Erica Valencic, Maura Bin, Emmanouil Athanasakis, Pio D’Adamo, Eirini Fragkiadoulaki, Gemma Calamandrei, Öykü Dinckol, Fabio Barbone, Luca Ronfani

**Affiliations:** 1 Institute for Maternal and Child Health - IRCCS “Burlo Garofolo”, Trieste, Italy; 2 Department of Medical and Biological Sciences, University of Udine and Institute of Hygiene and Clinical Epidemiology, University Hospital of Udine, Udine, Italy; 3 Department of Medical Sciences, University of Trieste, Piazzale Europa, Trieste, Italy; 4 Istituto Superiore di Sanità, Rome, Italy

**Keywords:** developmental disorder, mercury toxicity, heavy metal toxicity, schooling age

## Abstract

**Background:**

The extent to which prenatal low-level mercury (Hg) exposure through maternal fish intake and heavy metals exposure affect children’s neurodevelopment is controversial and may appear in the long term. In 2007, a prospective cohort, the Northern Adriatic Cohort II (NAC-II), was established to investigate the association between prenatal Hg exposure from maternal fish consumption and child neurodevelopment. The study enrolled 900 pregnant women, and 632 and 470 children underwent neurodevelopmental evaluation at 18 and 40 months of age, respectively. The NAC-II cohort is a part of the Mediterranean cohort in the “Public health impact of long-term, low-level, mixed element exposure in susceptible population strata” project.

**Methods:**

This protocol describes the follow-up assessment of the effects of prenatal low level Hg and other heavy metals exposure on the developing nervous system of the children born within the NAC-II who reached the age of 7 years. Child diet components are estimated through a Diet Diary. Child hair and urine are collected for determination of Hg level. In addition, levels of other potentially neurotoxic metals, namely Manganese, Cadmium, Lead, Arsenic, and Selenium, are also measured in the same matrices.

**Discussion:**

This protocol extends to the first years of schooling age the evaluation of the neurotoxicant effect of Mercury and of the other heavy metals on children’s neurodevelopment, adjusting for the potential confounders, such as the lifestyles and social economic status of children’s families. Longitudinal analysis of neurodevelopment, assessed in different ages (18 months, 40 months, and 7 years), are performed.

## BACKGROUND

Among different environmental pollutants Hg is proven to be highly toxic to humans, and its effects are mostly evident in the developing nervous system, especially if the absorption occurs in uterus and early in life.^1^^–^^3^

The major source of exposure and contamination is through the fish intake^4^; in particular, biomethylation of inorganic Hg through the bacteria in aquatic system produces methylmercury (MeHg).^5^ The rate of accumulation increases in bigger and older predatory fishes.^6^ After the ingestion of contaminated fish or seafood, 95% of MeHg is almost totally absorbed from the gastrointestinal tract, and in pregnant women, it crosses the placenta and blood brain barrier of the foetus, increasing the risk of neurodevelopmental delay.^1^^,^^7^^–^^13^ The developmental neurotoxicity of MeHg became evident after two major incidents occurred in Japan and in Iraq: many children showed symptoms, such as mental retardation, since the mothers’ diet during pregnancy was characterized respectively by intake of contaminated fish^14^^–^^16^ and grain.^17^

More recent studies focused on prenatal exposure to low dose of MeHg in populations with a frequent consumption of fish. The studies of Faroe Island and New Zealand indicated some kind of impairment in neurocognitive functions; conversely, a study in Seychelles Islands did not evidence an association between chronic low dose Hg or MeHg exposure and neurodevelopmental impairment.^18^^–^^22^

Despite the potential risk of low level of Hg contamination, fish and seafood are fundamental sources of essential nutrients, proteins, vitamins, minerals, and fatty acids,^23^^–^^25^ and consuming fish during early pregnancy can be beneficial to early infancy cognitive development.^26^ Many health agencies developed guidelines to encourage the fish consumption during the pregnancy while reducing the risk of exposure to Hg and potential toxic contaminants.^27^ A systematic review on the association between neurodevelopmental outcomes and maternal fish intake during pregnancy in some cohorts confirms the content of existing guidelines recommending fish consumption.^28^ However, literature is not sufficient to evaluate the type and quantity of fish consumption to recommend.^29^

To adequately evaluate the association between neurodevelopment and fish intake, some issues have to be considered: first, a detailed food frequency questionnaire is needed.^29^ Second, it is difficult to assess cognitive development in early infancy and childhood because infants show rapid and non-linear changes in nervous system development of mental and psychomotor functions, especially in the first years of life, and the results vary depending on time of evaluation and the effects of different environmental factors.^30^ In such a framework, repeated assessment from early infancy to school age is advisable to overcome individual differences in neuropsychological trajectories and detect delayed effects. To our knowledge, there are only few studies that consider different development steps reaching school-age.^9^^,^^20^^,^^30^^,^^31^ Third, an adequate and comprehensive adjustment for socio-demographic and environmental interrelated factors is needed. Genetic factors have also to be taken into account, since there can be a genetic predisposition to neurotoxicity of MeHg, even at low levels. Indeed, some polymorphisms have been associated to an increased susceptibility to MeHg, while other variants are able to protect against adverse effects.^32^^–^^39^ Finally, in addition to MeHg, other heavy metals (eg, manganese [Mn], arsenic [As], cadmium [Cd], lead [Pb], or their mixtures) affect in various ways brain and behavior development.^40^^–^^49^ Conversely, polyunsaturated fatty acids (PUFAs) and Selenium (Se) promote brain development and mitigate Hg toxicity.^8^^,^^50^ A comprehensive evaluation of exposures is needed to assess their effects on child neurodevelopment.

In 2007, the University of Udine (Italy) established a prospective cohort (NAC-II) in a coastal area of northeastern Italy with Hg pollution, to investigate the association between prenatal Hg exposure from maternal fish consumption and child neurodevelopment.^51^^,^^52^ The NAC-II cohort is a part of the Mediterranean cohort involved in the “Public health impact of long-term, low-level, mixed element exposure in susceptible population strata” project (PHIME). At 18 months, neurodevelopment of children enrolled in the NAC-II cohort was associated with maternal IQ and with child’s fish intake that appeared beneficial for cognitive development.^53^ Furthermore, some evidence of the association between THg and decreasing in developmental motor scores was found. Despite the large number of potential confounders taken into account, only a small proportion of the variability was explained, indicating that neurodevelopment is a multifactorial phenomenon and that residual confounding may exist in our estimated effects. Furthermore, these results should be confirmed at older ages.^54^

Further and more extensive evaluations at older ages could yield different results,^55^ and more sensitive and specific tests could be essential to determine the magnitude of effects. The main aim of this follow-up is to evaluate the neurocognitive development of the NAC-II cohort children at 7 years and to collect further information on the exposure to heavy metals, genetic variants and food habits. The assessment at this age allows for detecting specific learning disorders and attention difficulties.

## METHODS/DESIGN

### Participants

The NAC-II cohort was enrolled at the Institute for Maternal and Child Health - IRCCS Burlo Garofolo (Burlo) of Trieste (Italy), from April 2007 to April 2009. A detailed description of the study protocol and inclusion/exclusion criteria, have been published elsewhere.^51^^,^^52^ The study enrolled 900 pregnant women; 767 remained in the study at delivery, 632 children underwent neurodevelopmental evaluation using the Bayley Scales of Infant and Toddler Development Third Edition (BSID-III) at the age of 18 months, and 470 children were evaluated at 40 months. Descriptive statistics at baseline are shown in Table 1. The participants of the present study are 7 years old children (*n* = 632), whose parents gave informed consent to the follow-up carried out at the Neuropsychiatry Ward of the Burlo. Parents are contacted by phone to participate in the follow-up visit, which includes the child neuropsychological assessment, a 3-day diet diary (3-DDD), a recall questionnaire at 7 years old, and the collection of child biological samples.

**Table 1.** Descriptive statistics at delivery

**Sociodemographic characteristics of mothers and their children at delivery (baseline)**	**(*N* = 767)**	**(*N* = 632)**
Mother’s age at delivery, years, mean (SD) [median]	33.1 (4.4) [33]	33.3 (4.3) [33]

Maternal BMI before pregnancy, mean (SD) [median]	22.6 (3.8) [21.9]	22.7 (3.8) [22.1]

Weight gain during pregnancy, kg, mean (SD) [median]	13.4 (4.5) [13.0]	13.4 (4.5) [13.0]

Mother’s occupation, *n* (%)		

Employed on maternity	572 (75.8)	473 (75.9)

Employed worker	67 (8.9)	56 (9.0)

Housewife	68 (9.0)	51 (8.2)

Other condition	48 (6.4)	43 (6.9)

Mothers’ marital status, *n* (%)		

Married/Living together	683 (89.9)	565 (90.4)

Widow/single/never married/separated/divorcing	77 (10.1)	60 (9.6)

Mother’s educational level, *n* (%)		

Elementary and middle school	139 (18.2)	105 (16.7)

High school	364 (47.6)	304 (48.2)

University degree	262 (34.3)	221 (35.1)

Maternal Intelligence Quotient, Raven’s Progressive Matrices, mean (SD) [median]	118.2 (11.7) [123]	118.9 (11.2) [125]

Home size, *n* (%)		

<50 mq	53 (7.0)	46 (7.4)

50–100 mq	514 (67.8)	421 (67.5)

>100 mq	191 (25.2)	157 (25.2)

Number of cigarettes smoked, by mother, during pregnancy, mean (SD) [median]	170.9 (606.1) [0]	154.9 (584.3) [0]

Alcoholic drinks per week during pregnancy, number of glasses per week, mean (SD) [median]	1.5 (2.9) [0.3)	1.5 (2.8) [0.3]

Fish consumption of mother during pregnancy, 150-gram servings/week, mean (SD) [median]	1.5 (1.1) [1.1]	1.5 (1.1) [1.1)

Children’s sex, *n* (%)		

Male	385 (50.5)	314 (50.0)

Female	377 (49.5)	314 (50.0)

Birth weight, g, mean (SD) [median]	3,392.5 (466.3) [3,380.0]	3,404.2 (469.8) [3,397.5)

Birth length, cm, mean (SD) [median]	50.0 (2.1) [50.0)]	50.1 (2.2) [50.0]

SD, standard deviation.

The study was approved by the ethics committee of Burlo (CE/V-109-12/04/2010). Istituto Superiore di Sanità (ISS) collaborated to develop this protocol within the “Cross-Mediterranean Environment and Health Network” (CROME LIFE) project.

### Previously collected data

At enrolment (20–22 gestational week), a short questionnaire was administered to women to provide an assessment of parent’s socio-demographic information, maternal health, and food consumption habits. At 20–32 gestational week, women took the Raven’s Progressive Matrices Test.^56^

Detailed information about general health habits, family’s sociodemographics, and occupational information were collected in a long questionnaire filled in after the delivery.^51^ At the 18- and 40-month follow-up visits, supplementary questionnaires were completed detecting changes in sociodemographic information, socio-economic status, and child’s fish consumption; neurodevelopmental evaluation (BSID-III test) and assessment of family environment using Home Observation for Measurement of the Environment questionnaire (HOME)^51^^,^^57^ were done. Mothers’ hair, blood, urine, cord blood, and breast milk were collected in different phases of the study; concentrations of As, Cd, copper (Cu), Pb, Mn, Se, and zinc (Zn) were determined. Selected PUFAs were measured in maternal serum. Genotyping analyses were done.

Table 2 provides more details regarding the previous phases of the study.

**Table 2.** Questionnaires, test and biological samples collected before the 7 years old of children

**Previous phases of the study**	**Questionnaires, test or biological samples**	**Information collected**
**During pregnancy**	Short questionnaire	It was designed to identify any excluding conditions and to provide a quick assessment of parent’s socio-demographic information and maternal frequency of consumption of food items (eg, fish, vegetables) and smoking status.

	Raven’s Progressive Matrices Test	Evaluation of maternal intelligence.

	Maternal Hair	Measurement of: THg and MeHg.

	Maternal Urine	Measurement of THg.

	Maternal blood	Genotyping analysis. Measurement of: THg, MeHg, As, Se, Mn, Cu, Zn, Pb, Cd and PUFAs (from serum).

**At delivery**	Cord blood	Genotyping analysis. Measurement of: THg, MeHg, As, Se, Mn, Cu, Zn, Pb and Cd.

**Within the first month after delivery**	Long questionnaire	It was designed to collect: sociodemographic and health status information on mothers and their family, information on pregnancy and delivery and health status of the newborn child, a detailed residential and occupational history of the mother, maternal smoking, maternal drinking, and general dietary habits (through a detailed food frequency assessment of her consumption of 138 food items adapted from a validated food frequency questionnaire).

	Breast milk	Measurement of: THg, MeHg, As, Se, Mn, Cu, Zn, Pb and Cd.

**At 18 and at 40 months ** **of age of children**	Supplementary questionnaire	It assessed changes in residence, maternal marital and occupational status, anthropometric measures (weight and height) and developmental milestones of the child, breastfeeding history, child intake of fish, diseases, and day-care attendance.

	Bayley Scales of Infant and Toddler Development Third Edition (BSID-III)	Evaluation of child neurodevelopment.

**Between 18 and 40 months ** **of age of children**	Home Observation for Measurement of the Environment questionnaire (HOME)	Evaluation of family environment (ie, how parents and children interact in the home context)

### 7-year follow-up

### Neuropsychological outcome

Neuropsychological assessment is carried out by trained psychologists; children are assessed using the Wechsler Intelligence Scale for Children – Fourth Edition (WISC IV),^58^ and some subtests of NEuroPSYchological Assessment, (NEPSY II),^59^ MT Reading Test,^60^ an Italian standardized test for reading abilities, and 16 words test for writing abilities.^61^

During the assessment, mothers fill in the Child behaviour checklist (CBCL).^62^ An anthropometric evaluation of child is also carried out (Table 3).

**Table 3.** Questionnaire, test, and biological samples collected at the follow-up of 7 years old of children

**Questionnaires, test or biological samples**	**Information collected**
Wechsler Intelligence Scale for Children – Fourth Edition (WISC IV)^a^	Evaluation of the cognitive skills to assess intelligence and cognitive functions of children in the following composite areas: Verbal Comprehension, Perceptual Reasoning, Working Memory, Processing Speed, Full Scale IQ.

NEuroPSYchological Assessment (NEPSY II)^b^	Measurement of neuropsychological functions and dysfunctions in preschool and school-age children. We use only some tests regarding the Attention domain and Executive Functioning (Auditory Attention and Response Set, Inhibition).

MT Reading Test^c^	Evaluation of the reading skills (speed and accuracy) for early identification of specific Learning Disabilities.

Writing of 16 words	Evaluation of the writing skills. Participants are asked to write 16 words read aloud by examiner, an Italian standardized test to prevent possible writing difficulties.

Child behaviour checklist (CBCL)^d^	Assessment of “social competence” and “behaviour problems” to detect emotional and behavioural problems in children.

Recall Questionnaire	It was designed to update the information on: family’s sociodemographic, occupational history of parents, housing conditions and changes in residence, parental smoking and drinking, anthropometric measures (weight and height) and general daily care of the child, frequency of child’s fish consumption, child’s health history and child’s lifestyle and habits (involvement in extracurricular activities as sport, use of electronic device, etc).

3 Days Diet Diary	Assessment of dietary exposure in the child to fill in the week before the neurodevelopmental assessment.

Urine	Measurement of: THg, As, Cd, Mn, Pb and Se (ng/ml), creatinine, organophosphate and pyrethroid pesticides and other pesticides (ng/mL) in the spot urine collected the day before the neurodevelopmental assessment.

Hair	Measurement of: THg and Mn (ng/mg)

Saliva	Genotyping analysis. During the visit for the neuropsychological assessment the saliva samples are collected with Oragene DNA self-collection kit in those cases in which the umbilical cord tissue (collected at delivery in the first phase of the study) was not available.

^a^Wechsler Intelligence Scale for Children – Fourth Edition.

^b^NEuroPSYchological Assessment.

^c^Prove di Lettura MT (Memory and Transfert group Reading Test).

^d^Child Behaviour CheckList.

### Biological samples

Biological samples are collected and labelled with a unique identification code. Urine and hair are collected for the evaluation of metal levels, and saliva samples are collected for genotyping analysis.

Urine samples are collected in a 50 mL Hg-free tube (BD Falcon) and stored at −80°C within 24 hours from collection. As indicators of exposure, the level of THg, As, Cd, Mn, Pb, and Se, as well as organophosphate and pyrethroid pesticides, are considered.

For hair samples, the strands are gathered by cutting the hair close to the root in the occipital region of the scalp (equal to 200 mg). Each sample is stored at room temperature in a Hg-free, sealed plastic bag labelled until analysis. Levels of THg and Mn are evaluated in hair.

Saliva samples are collected in those cases in which the umbilical cord tissue was not available (Table 2). The Oragene DNA self-collection kit is used to assure maximum quantity of the saliva for genotyping purposes. The vials are stored at room temperature until the analysis. The umbilical cord tissue, already collected and stored in vials at −80° degrees (Table 2), are analyzed for genotyping purposes.

The saliva and hair samples are collected in the hospital when the children come for the neurodevelopmental assessment. Sample collection and storage are done according to a protocol developed by the Istituto Superiore di Sanità, and University of Udine and replicating the procedures already applied in the previous collection of biological samples.^51^ Measurement methods for metals are provided in eMaterials 1.

Genetic analysis includes the evaluation of polymorphisms that could modulate the detrimental effects of Hg at low exposure and other heavy metals. Whole Genome Genotyping is performed, and only polymorphisms based on current literature are analyzed.

### Tools for exposure measurement: evaluation of confounding elements

Mothers/caregivers fill in a “3-DDD”, evaluating the exact dietary intake, including types and amounts of foods consumed by the child in a 3-day period (2 week-days and 1 weekend day, not necessarily consecutive, in the week before the neurodevelopmental assessment). The diary data are analyzed with Microdiet software (V2.8.6, Downlee Systems Ltd., High, Peak, UK). The nutrient analysis is performed using the Italian food composition database.^63^

Socio-demographic characteristics, housing conditions, parents and child lifestyle, and health information are collected through a self-administered Recall Questionnaire. Table 3 shows the follow-up synthesis.

### Statistical analyses

Frequency distributions and summary measures of the following variables are computed: maternal IQ and marital status; housing conditions and socioeconomic indicators (house property and size, parent’s educational level, and occupational status); parent’s smoking habits and children’s exposure to passive smoking at home; children’s characteristics (gender, weight and height, hospital admission and cause, medications, and chronic illness) and life styles (fish consumption, physical activity, electronic device use); metal concentrations; and neurodevelopment scores. Metals’ concentrations are log_2_ transformed. Association between child neurodevelopment at 7 years and metal concentrations (with and without the log_2_ transformation) in biological samples are assessed through multiple linear regression and adjusted for the effect of potential confounders. Separate models are performed for each neurodevelopment score and each metal’s concentration. Stratified analysis by child’s gender are conducted to assess if the effect of metal’s concentration on neurodevelopment differed. SAS (version 9.4 SAS Institute INC., Cary, NC, USA) is used for analysis.

### Limitations

Cohort studies are sensitive to loss to follow-up of the participants enrolled for several reasons, including the children’s extracurricular commitments. However, at 18 and 40 months of age, a good rate (82%) and a quite good rate (69.5%), respectively, of mother-child pairs were in follow-up. Given that the study uses a range of neurocognitive tests, ensuring a deep evaluation of many neurodevelopmental aspects, parents are more motivated to come and participate. Further limitation is represented by the difficulty in biological sample collection: the hair length can be insufficient, and children can show some difficult in providing the proper amount of urine or saliva. In these cases, the researchers contact the family again to have another possibility of collection.

### Strengths

On the other side, this follow-up offers a number of strengths: the possibility to correlate the data collected at 7 years with those previously collected (during pregnancy, at delivery, and at 18 and 40 months). This is a great advantage over previous studies and allows for assessing the sensitivity of estimated associations between metals exposure and neurodevelopment, using concentrations in different samples and different timing and adjusting for confounding effects. Furthermore, this cohort allows for exploring gene-environment interactions in the toxicokinetics of metals and for measuring concentrations of a number of other neurotoxic and beneficial trace elements, as well as nutrients essential to the developing nervous system. This will enable us to control for their potential confounding effects on the association between mercury and neurodevelopment, thereby avoiding the imprecision and information biases that can affect estimates of nutrients.

## APPENDIX A. SUPPLEMENTARY DATA

eMaterials 1. Measurement methods and analytical quality control for metals
