# Supplementary material for: Impact of Methylmercury and Other Heavy Metals Exposure on Neurocognitive Function in Children Aged 7 Years: Study Protocol of the Follow-up
Source: J Epidemiol. 2021 Feb 5;31(2):157–63. doi: 10.2188/jea.JE20190284 (PMC7813770; doi:10.2188/jea.JE20190284)
Supplement: Supplementary file 1 [file je-31-157-s001.pdf]

eMaterials 1. Measurement methods and analytical quality control for metals

As, Cd, THg, Mn, Pb and Se in urine and and THg and Mn in hair were obtained with High Resolution-Inductively Coupled Plasma Mass Spectrometry (HR-ICPMS) (ElementII, Thermo Scientific, Bremen, Germany).[1,2]

Hair THg was determined using Direct Mercury Analyzer (DMA-80 Tricell, FKV) and hair Mn with ICP-MS.[3]

For each matrix, blanks and appropriate certified reference materials are included for all analytical runs; all analytical methods are accredited by the Italian Accreditation Institute (ACCREDIA). Limit of detection (LOD) was determined applying the 3σ criteria.

The analytical methods to measure organophosphate and pyrethroid pesticides in children urine are described by Garì et al. [4]

REFERENCES

1. Ruggieri F, Alimonti A, Bocca B. Full validation and accreditation of a method to support human biomonitoring studies for trace and ultra-trace elements. TrAC Trends Anal Chem. 2016;80:471–485.
2. Alimonti A, Bocca B, Ruggieri F. Metodo di riferimento per la determinazione degli elementi chimici in matrici biologiche umane: prestazioni analitiche e incertezza del dato (Reference method for the determination of chemical elements in human biological matrices: analytical performances and uncertainty of data). Roma: Istituto Superiore di Sanità; 2015. (Rapporti ISTISAN 15/30).

3. Domanico F, Forte G, Majorani C, et al. Determination of mercury in hair: comparison between gold amalgamation-atomic absorption spectrometry and mass spectrometry. J Trace Elem Med Biol. 2017;43:3–8.
4. Garí M, González-Quinteiro Y, Bravo N, et al. Analysis of metabolites of organophosphate and pyrethroid pesticides in human urine from urban and agricultural populations (Catalonia and Galicia). Sci. Total Environ. 2018;622-623:526-533.
